# Evaluation of oral care protocols practice by dentists in Rio de Janeiro towards HIV/AIDS individuals

**DOI:** 10.1186/s12903-020-0999-7

**Published:** 2020-01-14

**Authors:** Carina Maciel Silva-Boghossian, Brenda Azzariti Berrondo Boscardini, Claudia Maria Pereira, Edson Jorge Lima Moreira

**Affiliations:** School of Dentistry, Postgraduate Program in Dentistry, University of Grande Rio (UNIGRANRIO), Rua Prof. Jose de Souza Herdy, 1160, Jardim 25 de Agosto, CEP, Duque de Caxias, RJ 25071-202 Brazil

**Keywords:** Dental professional, Dentist, Oral health, HIV, AIDS

## Abstract

**Background:**

The aim of this study was to evaluate the dentists’ knowledge and practice regarding HIV positive individuals’ oral care in Rio de Janeiro State.

**Methods:**

Dentists from Rio de Janeiro State (*n* = 242) answered an electronic questionnaire on biosafety procedures, oral manifestations of AIDS, and knowledge of HIV infection. Collected information was stratified by gender, and data were analyzed using Chi-square and *t* tests.

**Results:**

From the 14 oral manifestations investigated, oral candidiasis, necrotizing ulcerative gingivitis, and hairy leucoplakia were more associated with HIV, with no differences between the responses from men and women. Above 85% of the participants would be concerned about becoming infected with HIV after a needle/ sharp object injury and more than 80% of them were willing to be tested for HIV. However, significantly more women (98.8%), compared to men (91.3%), said they knew that HIV/ AIDS patients can contaminate dental care professionals, *p* = 0.007. There was a significant difference in the answers for the questions: “Are there special dental clinics for treatment of HIV/AIDS patients in Brazil?” (*p* = 0.044), and “Do the negative HIV tests surely indicate that the persons are free of viruses?” (*p* = 0.005). Significant differences between men and women were also observed regarding use of disposable mask (*p* = 0.01), and cap (*p* < 0.0001).

**Conclusion:**

Most dentists who participated in the study presented a good knowledge on the care of HIV/ AIDS individuals, including biosafety protocols and in terms of the oral manifestations commonly associated to AIDS.

## Background

After decades of its discovery, the human immunodeficiency virus (HIV) infection is still an alarming health public problem [1, 2]. Infected individuals may develop the acquired immune deficiency syndrome (AIDS) when the infection reaches advanced stages [2]. In that condition, immunosuppression is measured through the evaluation of CD4+ lymphocytes as well as the counts of virus particles in carriers’ blood. Levels of CD4+ bellow 200 mg/ dL indicates deficient immune response, which is one important indicator for highly active antiretroviral therapy (HAART) initiation [3]. Due to HAART, currently, there are millions of people living with HIV in the world without AIDS symptoms [3–5]. However, non-symptomatic HIV+ individuals may still transmit the virus through sex without preservatives, shared needles, needle stick accident, pregnant women and even nursing mothers [5]. Therefore, routine screening tests for the identification of HIV infection is still necessary in order to avoid spreading of contamination [2].

Additionally, HIV+ individuals may have oral pathognomonic manifestations of AIDS, including oral candidiasis, hairy leucoplakia, Kaposi sarcoma, linear gingival erythema, necrotizing ulcerative gingivitis, necrotizing ulcerative periodontitis, and non-Hodgkin lymphoma [6–9]. Those oral lesions may work as an indicator of infection progression [6–8]. Therefore, oral cavity clinical signs become extremely important for the primarily presumption for HIV infection [10]. In fact, dental surgeons may be the first health professionals to suspect of positivity [11].

Furthermore, during dental treatment, there is a considerable risk of cross-contamination [12]. Cross-infection may occur by accident with cutting or puncture contaminated material, for example, needle perforation [13]. Dental treatment sets are a perfect candidate place to occur cross-contamination due to saliva and blood sprays produced during treatment. However, it is worth to mention that the risk of HIV infection in that environment is reported to be very low, around 0.3% [14, 15]. Nonetheless, dentists must be well prepared to provide adequate attention to the increasing number of people living with HIV. In this scenario, it includes accurate diagnosis and treatment plan conducted under conditions that allow infection control [16]. Currently, there is no available information on dentists from Rio de Janeiro State on their knowledge on the care of individuals living with HIV/ AIDS. Thus, the aim of this study was to evaluate the dental surgeons’ knowledge and practice regarding patient care towards HIV positive individuals.

## Methods

This cross-sectional study was carried out from January to July 2018. Electronic questionnaire was developed in Google Forms, containing 47 questions, which included questions about dental care for HIV+ patients. The participants were dental surgeons regularly register in the Regional Council of Dentistry of Rio de Janeiro state (CRO-RJ). This study was submitted and approved by the Research Ethics Committee of Unigranrio (# 2335672). Participants’ consent was obtained automatically after they had read the Informed Consent form and agreed to access the survey by clicking on the link to the study questionnaire.

The electronic questionnaire was sent to about 6000 professionals registered at CRO-RJ to their email addresses. Details of the four parts of the questionnaire can be found in a previous work [17]. Furthermore, an English version of the questionnaire can be found as Additional file 1.

### Data analysis

All statistical tests used in the present study were performed with a statistical program (SPSS Statistics 20, IBM Brazil, São Paulo, Brazil). The distribution of specific responses on HIV+ patients were analyzed by grouping by gender. The tests used to evaluate significant differences between groups were t-test for independent samples and Chi-square. The level of significance established for all analyzes was 5%.

## Results

Table 1 presents demographic characteristics of the participants (*n* = 242). The majority of participants were female (*n* = 162; 67%). Women (37.9 ± 10.7 years) were significantly younger than men (42.9 ± 12.1; *p* = 0.001, T test). Professionals with a previous biomedical education other than Dentistry were 6.8% of women and 5% of mean. A significant difference in years of graduation from university was found between women 14.5 (± 11.3) and men 18.4 (± 11.1), *p* = 0.012. Several participants had more than one dental specialization among the 21 areas cited. This accounted for 16% of women and 21.3% of men (*p* = 0.024; Chi-square test). Most of the participants, 67.1% of women and 61.3% of men, worked in only one dental clinic.

**Table 1:** Demographic characteristics of the study participants according to gender

Variables	Female (*n* = 162)	Male (*n* = 80)	*P* value
Mean age in years (± standard-deviation)	37.9 (10.7)	42.9 (12.1)	0.001^**†**^
Degree in another biomedical profession (%yes)	6.8	5	> 0.05
Years of graduation from university (±standard-deviation)	14.5 (11.3)	18.4 (11.1)	0.012^**†**^
*Participants’ specialty*			
• CBMF	0.6	10	0.024*
• Restorative dentistry	1.9	2.5	
• DTM	0.6	0	
• Endodontics	11.7	7.5	
• Estomatology	1.2	1.3	
• Dental service management	0	1.3	
• Implant dentistry	4.9	3.8	
• Geriatric dentistry	0.6	0	
• Work dentistry	0	1.3	
• Hospital dentistry	0.6	1.3	
• Legal dentistry	0.6	1.3	
• Pediatrics dentistry	14.2	3.8	
• Orthodontics	10.5	5	
• Oral pathology	0	1.3	
• Periodontics	9.3	8.8	
• PNE	0.6	0	
• Dental prosthesis	3.7	11.3	
• Oral radiology	0.6	1.3	
• Family health	3.1	1.3	
• Public health	0.6	1.3	
• Semiology	0.6	0	
• More than one specialty	16	21.3	
• No specialty	17.9	15	
*Work location (public or private service)*			
• Only one	67.1	61.3	> 0.05
• More than one	31.1	37.5	
• Do not work in clinical settings^‡^	1.9	1.3	

*Chi-square test; ^**†**^ T test for independent samples; ^‡^ work in education, for example; *CBMF* oral and maxillofacial surgery, *DTM* temporomandibular joint dysfunction, *NS* non-significant, *PNE* special needs patients

Most participants answered ‘yes’ to the question ‘Can HIV/AIDS individuals be diagnosed with oral lesions?’, accounting for 82.7% of women and 75% of men (Table 2). Table 2 is also presenting participants’ responses when they were asked if a list of oral manifestations were associated to HIV. Most part of answers were positive (‘yes’) for Kaposi’s sarcoma (89.5% of women and 90% of men), oral candidiasis (85.2% of women and 82.5% of men), hairy leukoplakia (63% of women and 60% of men) and necrotizing ulcerative gingivitis (71% of women and 62.5% of men). On the other hand, positive answers were below 50% for the remaining oral manifestations investigated. There was no significant difference in the distribution of answers between women and men.

**Table 2:** Distribution of answers on the professionals’ knowledge regarding oral manifestations in HIV/ AIDS individuals

Oral manifestations	% answer						*P* value
	Women (*n* = 162)			Men (*n* = 80)			
	Yes	No	Do not know	Yes	No	Do not know	
Can HIV/AIDS individuals be diagnosed with oral lesions?	82.7	15.4	1.9	75	22.5	2.5	> 0.05
Are those manifestations related to HIV/AIDS?							
• Kaposi’s sarcoma	89.5	6.2	4.3	90	8.8	1.3	> 0.05
• Oral candidiasis	85.2	14.2	0.6	82.5	12.5	5	> 0.05
• Hairy leukoplakia	63	20.4	16.7	60	26.3	13.8	> 0.05
• Periodontitis	43.2	49.4	7.4	40	55	5	> 0.05
• Necrotizing ulcerative gingivitis	71	21.6	7.4	62.5	28.8	8.8	> 0.05
• Herpes simplex	48.8	45.1	6.2	48.8	47.5	3.8	> 0.05
• Major aphthous	41.4	47.5	11.1	28.8	60	11.3	> 0.05
• Gingivitis	36.4	54.3	9.3	33.8	63.8	2.5	> 0.05
• Cytomegalovirus	44.4	25.3	30.2	51.3	31.3	17.5	> 0.05
• Herpes Zoster	46.3	42	11.7	48.8	46.3	5	> 0.05
• Salivary gland infection	25.9	53.1	21	25	61.3	13.8	> 0.05
• Lichen planus	33.3	48.8	17.9	30	58.8	11.3	> 0.05
• Condiloma	38.9	38.9	22.2	37.5	45	17.5	> 0.05
• Xerostomia	29.6	56.8	13.6	21.3	70	8.8	> 0.05

*Chi-square test; *NS* non-significant

Table 3 shows the distribution of answers related to professionals’ knowledge on biosafety and the care of HIV+ individuals. A significant number of professionals were worried about acquiring the virus after an accident with a contaminated sharp object (87.7% of women and 85% of men). Most professionals would be willing to be tested for HIV after the accident (82.7% of women and 80% of men). The majority have also said that dental professionals can be intermediate in the transmission of HIV (83.3% of women and 88.8% of men). Additionally, the majority said that medical care professionals are more prone to cross-infection related to HIV (98.8% of women and 91.3% of men). Only 19.1% of women and 27.5% of men said that the HIV tests present a 100% specificity.

**Table 3:** Distribution of professionals’ answers on biosafety and dental care of HIV+ patients

Questions	Distribution of answers (%)						*P* value*
	Women (*n* = 162)			Men (*n* = 80)			
	Yes	No	Do not know/ Maybe	Yes	No	Do not know/ Maybe	
After accident with a sharp object:							
- Would you be worried to be infected?	87.7	2.5	9.7	85	1.3	13.8	> 0.05
- Would you be tested for HIV?	82.7	3.1	14.2	80	2.5	14.6	> 0.05
Can dental professionals act as an intermediary for transmission of HIV?	83.3	14.2	2.5	88.8	10	1.3	> 0.05
Can patients with HIV/ AIDS contaminate dental professionals?	98.8	1.2	0	91.3	8.8	0	0.007
Can needle stick injury transmit HIV?	96.3	2.5	1.2	97.5	0	2.5	> 0.05
Are medical professionals more prone to cross-contamination?	82.7	14.8	2.5	85	8.8	6.3	> 0.05
Can saliva be a vehicle for the transmission of AIDS?	23.5	75.3	1.2	20	76.3	3.8	> 0.05
Is there a lot of HIV particles in the saliva of HIV/AIDS patients?	14.2	64.8	21	15	66.3	18.8	> 0.05
Is hepatitis B more communicable than HIV/AIDS?	81.5	9.3	9.3	86.3	5	8.8	> 0.05
Can CPR in patients with AIDS transmit HIV infection?	12.3	71.6	16	17.5	76.3	6.3	> 0.05
Do infection control methods for hepatitis B provide adequate protection against the transmission of HIV?	77.8	16	6.2	73.8	18.8	7.5	> 0.05
Are there special dental clinics for treatment of HIV/AIDS patients in Brazil?	22.8	19.8	57.4	35	10	55	0.044
Do all sterilization methods have cidal effects against HIV?	59.9	36.5	4.9	70	23.8	6.3	> 0.05
Can HIV be transmitted through aerosols by handpieces?	19.1	61.7	19.1	21.3	62.5	16.3	> 0.05
Do the negative HIV tests surely indicate that the persons are free of viruses?	4.9	92	3.1	16.3	83.8	0	0.005
Is Western blot a definite test for HIV/AIDS diagnosis?	26.5	28.4	45.1	40	21.3	38.8	> 0.05
Is ELISA a screening test for HIV infection?	83.3	3.7	13	76.3	6.3	17.5	> 0.05
Is the specificity of the HIV tests 100%?	19.1	61.1	19.8	27.5	52.5	20	> 0.05

* Chi-square test. *CPR* cardiopulmonary resuscitation

Table 4 presents the frequency of use of physical barriers according to gender. Although both groups do use mask with all patients (98.8% of women; 92.5% of men), this was significantly different between them (*p* = 0.01). Most of the women (92%) wear a cap during work, which was significantly higher than men (48.8%; *p* < 0.0001). In terms of other means of physical protection (protection goggles; 1 pair of gloves that is changed between patients; 2 pairs of gloves that are changed between patients; autoclaved handpiece; disposable gown; and plastic wrap), no statistical difference was detected between genders.

**Table 4:** Responses to measures of infection control of HIV transmission according to gender

Gender	Physical barrier	Frequency of use (%)				
		Use with all patients	Use with some patients	Use just for some procedures	Do not use	Others
Women (*n* = 182)	Mask *	98.8	1.2	0	0	–
	Protection goggles	69.8	6.2	13	7.4	3.7^**†**^
	Cap **	92	3.1	3.7	1,2	–
	1 pair of gloves - change between patients	99.4	0.6	0	0	–
	2 pairs of gloves - change between patients	21.3	10.6	11.9	56.3	–
	Autoclaved handpiece	44.4	6.8	29.6	19.1	
	Disposable gown	40.1	4.9	30.9	24.5	–
	Plastic wrap	84	3.1	4.9	8	–
Men (*n* = 80)	Mask *	92.5	0	2.5	3.8	–
	Protection goggles	60	8.8	23.8	7.5	0
	Cap **	48.8	5	21.3	25	–
	1 pair of gloves - change between patients	98.8	0	1.3	0	
	2 pairs of gloves - change between patients	29.5	7.7	7.7	55.1	–
	Autoclaved handpiece	52.5	10	22.5	15	
	Disposable gown	43.8	8.8	33.8	13.8	–
	Plastic wrap	78.8	7.5	8.8	5	–

*p = 0.01, and ** *p* < 0.0001, Chi-Square test between genders. ^**†**^ Wear prescription glasses

**Fig. 1:**
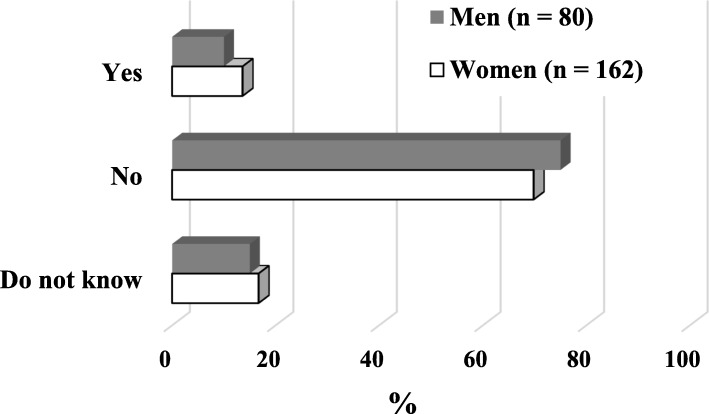
Answers to “Is AIDS now the most important health problem in the world?”

Most respondents answered “No” to the question “Now, is AIDS the most important health problem in the world?” (75% of women and 69.8% of men) (Fig. 1).

## Discussion

AIDS still figures among the ten major and most important world health problems, despite all progress in the studies focusing on the virus and transmission prevention. Therefore, it is imperative that dental professionals have all possible knowledge towards HIV+ patients’ care, mainly when it comes to cross contamination and pathogen dissemination. Having that in mind, the aim of the current investigation was to evaluate the level of dental care professionals’ knowledge and practice in relation to HIV/AIDS individuals in the State of Rio de Janeiro, Brazil.

At the beginning of this study, it was possible to reach about 5000 dentists through their e-mails registered at the state dental council of Rio de Janeiro. However, only 242 of those professionals accepted to participate in the study, representing a low response rate (4.8%). Nonetheless, it is still a comparable sample size to other studies [13, 18, 19]. In the study of Maia et al. [13], 170 dentists from Northeast Brazil were included; in Senna et al. [18], 140 dentists answered a questionnaire in another state capital. In the current investigation, most participants were women (66.9%). A predominance of female participants (64.3%) was also reported in other studies [18, 19].

More than 75% of participants know that HIV/ AIDS individuals can be diagnosed with oral manifestations. Moreover, most participants said that Kaposi’s sarcoma (89.5% of women and 90% of men) and oral candidiasis (85.2% of women and 82.5% of men) are oral signs of HIV/ AIDS. Those diseases seem to be the most known by dentists as related to HIV/ AIDS, as it was demonstrated by other studies [12, 20, 21]. In Oliveira et al. [20], 92.5 and 90.3% of participants said that Kaposi’s sarcoma and oral candidiasis, respectively, are oral manifestations of HIV/ AIDS. Similarly, Oberoi et al. [21] presented a percentage of “yes” of 95% for oral candidiasis, 86% for Kaposi’s sarcoma, 88% for necrotizing ulcerative gingivitis, 85% for hairy leukoplakia, 81% for Herpes zoster and major aphthous and 75% for salivary gland infection. In Sadeghi and Hakimi [12] work, several lesions were pointed out as associated to HIV/ AIDS. Interestingly, despite oral herpes simplex being an oral pathognomonic marker of HIV, current results showed a low rate of positive answers relating these lesions to HIV+.

Regarding accident with sharp objects, above 85% of the participants answered that HIV is transmitted via needle stick injury, which is in accordance with previous reports with dental students [10, 12, 17, 22, 23]. Nevertheless, this notion is not universal, as demonstrated by Aggarwal and Panat [24]. In the study of Rostamzadeh et al. [19] only 84% of the dentists affirmed that HIV/ AIDS prophylaxis is recommended after a needlestick injury.

Additionally, current data demonstrated that nearly 100% of individuals were worried of acquiring HIV after sharp object accident and would be taking a test afterwards. Those findings are a reflection of a great awareness on the existing risk in the injury with contaminated sharp objects [21, 25]. However, in another study [20], only 48% of participants said that they would be tested for HIV after a sharp object accident. Despite that low rate of concern with accidents, in Maia et al. [13] dentists presented major concerns in terms of work conditions in the care of HIV/ AIDS individuals. In that study, it was demonstrated that dentists who had continuous education towards HIV/ AIDS individuals’ care might feel more confident to provide better care. Interestingly, a Canadian study showed that people living with HIV/ AIDS would prefer to be treated by dentists who are knowledgeable on the condition and with previous experience in treating carriers [26]. In fact, participants of that study believed that an increase in the knowledge and in the clinical experience may create an improved relationship between the patient and the professional.

In the current study, it was shown that disposable masks are used by almost every participant. The use of cap was significantly different between men and women. This could be explained by the fact that women are more worried with their hair contamination when there is a contaminated spray produced during handpiece use. In terms of using protection goggles, the current data is much higher to the ones reported by Oliveira et al. [20], in which 8% of women and 21% of men used it with all patients. Overall, current findings showed that the use of individual protection equipment were more frequent when compared to another study [19].

In general, current participants consider AIDS, nowadays, as not the most important health problem in the world. It is in accordance with other studies, in which 68% [12] and 65% [24] of participants had similar answers. It is a fact that worldwide efforts on implementation of HAART have guaranteed longevity and quality of life to the ones living with HIV/ AIDS. Therefore, currently, it is not the most important health problem in the world. Nonetheless, it still is among the 10 major health problems in the world [5].

Not only is preventive approach a must in daily dental clinics, but it is also essential that dentists may be able to provide incipient diagnoses through the evaluation of oral cavity manifestations of HIV/AIDS. Ultimately, a good oral health will contribute for a good quality of life.

## Conclusion

Most dentists who participated in the study presented a good knowledge on the care of HIV/ AIDS individuals, including biosafety protocols and in terms of the most common oral manifestations associated to AIDS.

## Supplementary information

**Additional file 1.** Questionnaire. Adaptation of the online questionnaire created on Google Forms and employed in the study.

## Data Availability

The datasets used and/or analyzed during the current study are available from the corresponding author on reasonable request.

## Abbreviations

AIDS: Acquired immune deficiency syndrome
HAART: Highly active antiretroviral therapy
HIV: Human immunodeficiency virus
