# Supplementary material for: Evaluation of oral care protocols practice by dentists in Rio de Janeiro towards HIV/AIDS individuals
Source: BMC Oral Health. 2020 Jan 14;20:13. doi: 10.1186/s12903-020-0999-7 (PMC6961293; doi:10.1186/s12903-020-0999-7)
Supplement: Supplementary file 1 — Additional file 1. Questionnaire. Adaptation of the online questionnaire created on Google Forms and employed in the study. [file 12903_2020_999_MOESM1_ESM.docx]

**Adaptation of the online questionnaire created on Google Forms and employed in the study**

Gender ( ) female ( ) male

Age ________

How long have you been graduated? _________

Do you work in a public or private clinic, in hospital, or in a private dental office? _____________________________________________

What is your specialty in Dentistry? _________________________

Do you have previous degree in other health profession? What is it? _______________

After accident with a sharp object, would you be worried to be infected?

( ) yes ( ) no ( ) maybe

After accident with a sharp object, would you be tested for HIV?

( ) yes ( ) no ( ) maybe

Regarding the use of disposable mask:

( ) change it between patients; ( ) wear it with all patients; ( ) only wear it with some patients; ( ) only wear it during some procedures; ( ) do not wear it; ( ) other.

Regarding the use of protection goggles: ( ) wear it with all patients; ( ) only wear it with some patients; ( ) only wear it during some procedures; ( ) do not wear it; ( ) other.

Regarding the use of cap: ( ) change it between patients; ( ) wear it with all patients; ( ) only wear it with some patients; ( ) only wear it during some procedures; ( ) do not wear it; ( ) other.

Regarding the use of procedure gloves -1 pair: ( ) change it between patients; ( ) wear it with all patients; ( ) only wear it with some patients; ( ) only wear it during some procedures; ( ) do not wear it; ( ) other.

Regarding the use of procedure gloves -2 pairs: ( ) change it between patients; ( ) wear it with all patients; ( ) only wear it with some patients; ( ) only wear it during some procedures; ( ) do not wear it; ( ) other.

Regarding the use of autoclaved handpiece: ( ) wear it with all patients; ( ) only wear it with some patients; ( ) only wear it during some procedures; ( ) do not wear it; ( ) other.

Regarding the use of disposable gown: ( ) wear it with all patients; ( ) only wear it with some patients; ( ) only wear it during some procedures; ( ) do not wear it; ( ) other.

Regarding the use of plastic wrapping: ( ) change it between patients; ( ) wear it with all patients; ( ) only wear it with some patients; ( ) only wear it during some procedures; ( ) do not wear it; ( ) other.

Are the following clinical conditions considered oral manifestations of HIV/AIDS?

Kaposi’s sarcoma ( ) yes ( ) no ( ) do not know

Oral candidiasis ( ) yes ( ) no ( ) do not know

Hairy leukoplakia ( ) yes ( ) no ( ) do not know

Periodontitis ( ) yes ( ) no ( ) do not know

Necrotizing ulcerative gingivitis ( ) yes ( ) no ( ) do not know

Herpes simplex ( ) yes ( ) no ( ) do not know

Major aphthous ( ) yes ( ) no ( ) do not know

Gengivitis ( ) yes ( ) no ( ) do not know

Cytomegalovirus ( ) yes ( ) no ( ) do not know

Herpes Zoster ( ) yes ( ) no ( ) do not know

Salivary gland infection ( ) yes ( ) no ( ) do not know

Lichen planus ( ) yes ( ) no ( ) do not know

Condiloma ( ) yes ( ) no ( ) do not know

Xerostomia ( ) yes ( ) no ( ) do not know

Can patients with HIV/ AIDS contaminate dental professionals? ( ) yes ( ) no ( ) do not know

Can HIV/AIDS individuals be diagnosed with oral lesions? ( ) yes ( ) no ( ) do not know

Can dental professionals act as an intermediary for transmission of HIV? ( ) yes ( ) no ( ) do not know

Can needle stick injury transmit HIV? ( ) yes ( ) no ( ) do not know

Is hepatitis B more communicable than HIV/AIDS? ( ) yes ( ) no ( ) do not know

Are medical professionals more prone to cross-contamination? ( ) yes ( ) no ( ) do not know

Do the negative HIV tests surely indicate that the persons are free of viruses? ( ) yes ( ) no ( ) do not know

Is Western blot a definite test for HIV/AIDS diagnosis? ( ) yes ( ) no ( ) do not know

Is ELISA a screening test for HIV infection? ( ) yes ( ) no ( ) do not know

Is the specificity of the HIV tests 100%? ( ) yes ( ) no ( ) do not know

Can saliva be a vehicle for the transmission of AIDS? ( ) yes ( ) no ( ) do not know

Do infection control methods for hepatitis B provide adequate protection against the transmission of HIV? ( ) yes ( ) no ( ) do not know

Are there special dental clinics for treatment of HIV/AIDS patients in Brazil? ( ) yes ( ) no ( ) do not know

Is there a lot of HIV particles in the saliva of HIV/AIDS patients? ( ) yes ( ) no ( ) do not know

Can cardiopulmonary resuscitation in patients with AIDS transmit HIV infection? ( ) yes ( ) no ( ) do not know

Do all sterilization methods have cidal effects against HIV? ( ) yes ( ) no ( ) do not know

Is AIDS now the most important health problem in the world? ( ) yes ( ) no ( ) do not know

Can HIV be transmitted through aerosols by handpieces? ( ) yes ( ) no ( ) do not know
